# Semen levels of matrix metalloproteinase (MMP) and tissue inhibitor of metalloproteinases (TIMP) protein families members in men with high and low sperm DNA fragmentation

**DOI:** 10.1038/s41598-018-37122-4

**Published:** 2019-01-29

**Authors:** Larissa Berloffa Belardin, Mariana Pereira Antoniassi, Mariana Camargo, Paula Intasqui, Renato Fraietta, Ricardo Pimenta Bertolla

**Affiliations:** 1 Department of Surgery, Division of Urology, Universidade Federal de São Paulo, São Paulo, Brazil; 2 Hospital São Paulo, São Paulo, Brazil

**Keywords:** Medical research, Urology

## Abstract

Matrix Metalloproteinases (MMPs) and their regulators – Tissue Inhibitors of Matrix Metalloproteinases (TIMPs) – participate in extracellular matrix remodeling, fibrosis, and semen liquefaction, as well as to inflammatory activity. Seminal plasma has been shown to contain MMPs (MMP-2 and MMP-9) and TIMPs (TIMP-1 and TIMP-2). Also, a link between MMPs gene expression and excessive reactive oxygen species (ROS) has been established. In semen, ROS are associated with altered sperm function and increased DNA fragmentation. In this study, it is hypothesized that seminal MMPs and TIMPs levels are associated with sperm DNA fragmentation due to the fact that MMPs have been associated with semen quality. We also hypothesized that these proteins could predict DNA fragmentation status in sperm. Therefore, this study set out to verify if sperm DNA fragmentation levels relate to seminal levels of members of the MMP and TIMP protein families. The High sperm DNA fragmentation group presented lower seminal plasma levels of MMP-2, MMP-7, TIMP-1, TIMP-2 and TIMP-4 when compared to Low sperm DNA fragmentation group. Also, samples in the high sperm DNA fragmentation group presented higher acrosome integrity and lower mitochondrial activity levels when compared to low sperm DNA fragmentation samples. In the logistic regression analysis, MMP-2, MMP-7, and TIMP-4 classified samples as low and high sperm DNA fragmentation, with an overall model fit of 74.5%. Results from this study may demonstrate a specific inflammatory mechanism in samples with high sperm DNA fragmentation. This, in turn, can lead to the development of new studies regarding this mechanism and, in the future, create an opportunity to treat these patients for sperm DNA fragmentation by treating inflammatory seminal activity.

## Introduction

Matrix metalloproteinases (MMPs) are important constituents of ejaculated semen. These proteins belong to a group of proteolytic zinc-dependent enzymes (endopeptidases), which, alongside their inhibitors – tissue inhibitors of metalloproteinases (TIMPs) – participate in tissue restructuring by remodeling of the extracellular matrix^1–3^. Moreover, MMPs and other proteases (such as prostate-specific antigen – PSA) are involved in semen liquefaction^4^, in the female reproductive tract. Semen liquefaction is a necessary step for further sperm processes related to fertilization, such as capacitation^5^. MMPs have also been shown to affect sperm differentiation and morphological modifications^6^. Finally, MMPs interaction with sperm proteins has been associated with sperm viability, capacitation, and fertilization^7^.

TIMPs inhibit MMPs by forming a 1:1 molecular complex. Their expression has been demonstrated in the human testis and the seminiferous epithelium^8–11^. MMP-9 and TIMP-2 DNA polymorphisms are associated with decreased sperm concentration, morphology, and progressive motility^12^, and MMP-9 expression is higher in childless men when compared to normozoospermic fertile men^13^. Moreover, Pro-MMP-9 and MMP-9 levels are elevated in canine samples with low sperm counts^1^. Finally, MMPs and TIMPs modulate the inflammatory state in a number of tissues, such as lung, liver, and heart^14–20^.

A previous study from our group identified ELSPBP1 protein (Uniprot Accession Q96BH3) increased in sperm of patients with higher sperm DNA fragmentation. This protein is transferred to dead spermatozoa in bovine epididymides^21^. Characteristically, it presents four fibronectin type II (FN2) domains^21^. It is noteworthy that MMPs also present FN2 domains, and have also been associated with sperm functional quality^1,12,13^. We therefore hypothesized that proteins from the MMP and TIMP families, which participate in extracellular matrix remodeling by means of their FN2 domains are associated with sperm functional quality. In order to test this hypothesis, seminal plasma levels of MMP-1, MMP-2, MMP-7, MMP-9, and MMP-10, and all TIMPs (TIMP-1, TIMP-2, TIMP-3, and TIMP-4) were assessed in patients with low and high sperm DNA fragmentation.

## Results

### Semen and sperm functional analysis in high and low sperm DNA fragmentation samples

Results regarding semen and sperm functional analyses of men with low and high sperm DNA fragmentation are presented in Table 1. The patients were classified into “high” and “low” DNA fragmentation groups – all with semen within the 95th percentile values of fertile men, as per WHO guidelines^22^. We have previously performed this approach for defining groups of high and low sperm functional integrity^23–25^ and oxidative stress^26^. The minimum and maximum values of Comet Distributed Moment variable for low sperm DNA fragmentation were 0.86 and 29.09 (arbitrary units – a.u.) and for high sperm DNA fragmentation were: 47.66 and 94.16 (a.u.), respectively.

**Table 1:** Seminal and sperm functional analyses of men with low and high sperm DNA fragmentation. Groups were compared by Mann-Whitney test (values expressed in median; interquartile range).

	Low (n = 40)	High (n = 38)	*p*
Age (years)	34.0; 7.25	34.3; 13.75	0.128
Volume (mL)	3.3; 1.90	3.3; 1.67	0.579
pH	8.0; 0.50	8.0; 0.50	0.316
Liquefaction time (minutes)	27.5; 20.00	27.5; 21.25	0.354
Sperm concentration (x10^6^/mL)	100.8; 123.15	60.55; 77.77	0.583
Total count (x10^6^)	302.5; 461;25	301.7; 297.70	0.657
Progressive motility (%)	50.5; 10.75	54.0; 13.75	0.753
Non-progressive motility (%)	6.0; 3.75	4.5; 2.00	0.511
Immotile (%)	45.0; 12.75	41.0; 16.00	0.565
Morphology (% normal)	7.0; 4.75	6.5; 4.25	0.751
Round cells (x10^6^/mL)	0.9; 2.80	0.8; 4.60	0.503
Neutrophils (x10^6^/mL)	0.0; 0.27	0.0; 0.00	0.391
Comet distributed moment (a.u.)	25.7; 4.99	62.1; 15.50	<0.0001*
Acrosome integrity (%)	72.5; 13.00	78.5; 14.00	0.017*
DAB I (%)	11.0; 10.55	10.4; 13.87	0.980
DAB II (%)	63.5; 16.50	68.0; 22.62	0.686
DAB III (%)	14.0; 9.75	10.75; 10.75	0.023*
DAB IV (%)	7.0; 8.75	8.5; 14.12	0.086

*Statistically significant difference. High – high sperm DNA fragmentation group. Low – low sperm DNA fragmentation group.

a.u. – arbitrary units.

*Statistically significant differente (p < 0.05).

Results regarding semen and sperm functional analyses of men with low and high sperm DNA fragmentation are presented in Table 1. Conventional semen quality, as assessed by 2010 World Health Organization guidelines^22^, was comparable between groups. The high sperm DNA fragmentation group presented higher acrosome integrity and lower DAB class III levels, when compared to the low DNA fragmentation group.

### TIMPs and MMPs levels in high and low sperm DNA fragmentation samples

**Figure 1:**
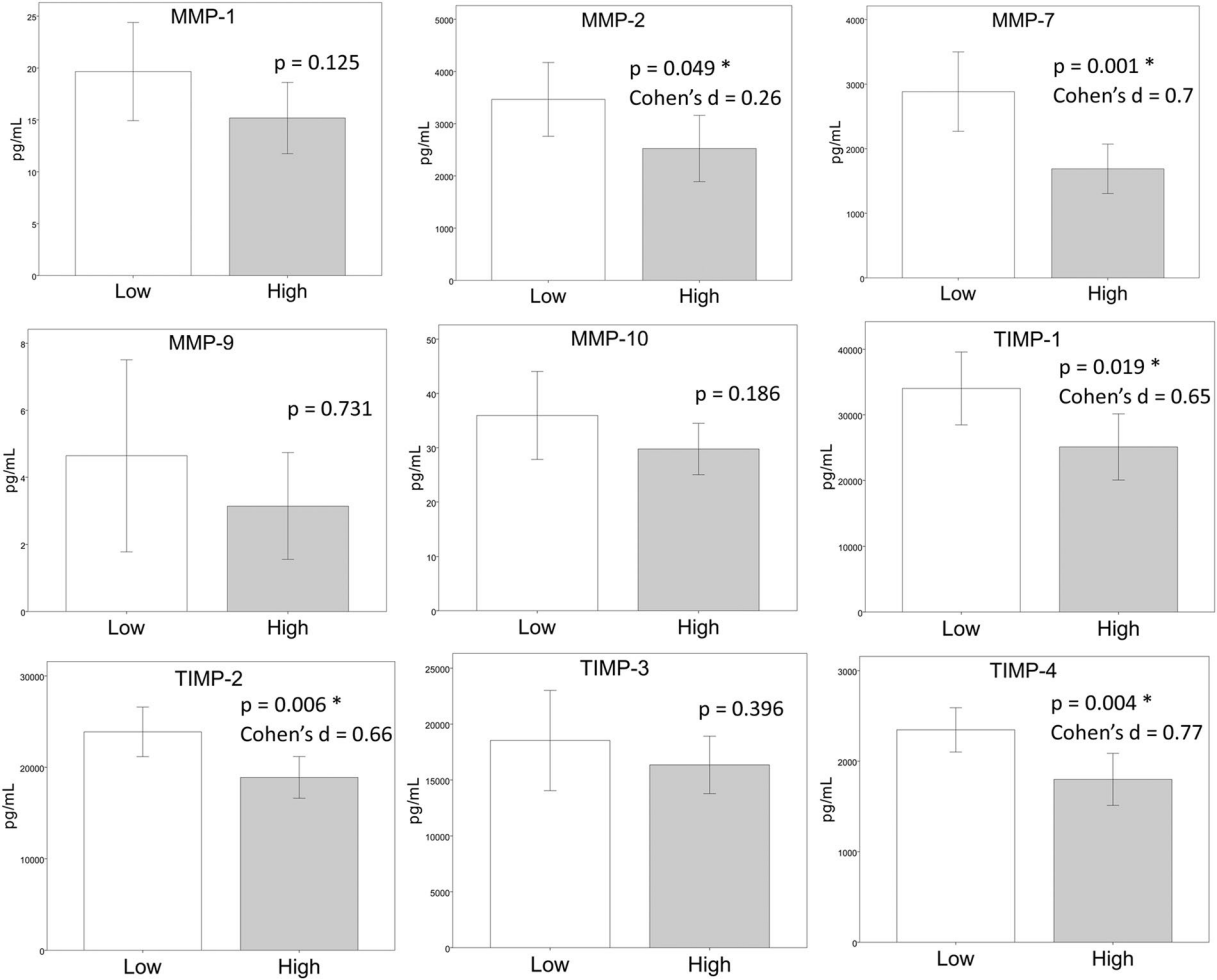
Bar graphs of MMP-1 (**A**), MMP-2 (**B**), MMP-7 (**C**), MMP-9 (**D**), MMP-10 (**E**), TIMP-1 (**F**), TIMP-2 (**G**), TIMP-3 (**H**) and TIMP-4 (**I**) levels in seminal plasma of men with high and low sperm DNA fragmentation. Groups were compared by a Student’s T test. The error bars indicate the confidence interval of 95% of the mean.

The high sperm DNA fragmentation group presented lower seminal levels of MMP-2, MMP-7, TIMP-1, TIMP-2, and TIMP-4 when compared to the low sperm DNA fragmentation group (control group) (Fig. 1). The effect size was low to medium for MMP-2 (Cohen’s *d* = 0.262) and medium to high for MMP-7 (Cohen’s *d* = 0.700), TIMP-1 (Cohen’s *d* = 0.653), TIMP-2 (Cohen’s *d* = 0.660) and TIMP-4 (Cohen’s *d* = 0.770).

**Figure 2:**
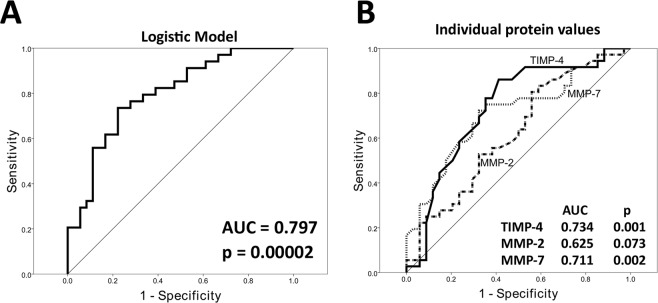
Receiver operating characteristic (ROC) curve showing the achieved sensitivity and specificity of the logistic regression model (**A**), and the sensitivity and specificity of MMP-2, MMP-7 and TIMP-4 in detecting high versus low sperm DNA fragmentation.

In a logistic regression model, proteins MMP-2, MMP-7, and TIMP-4 were predictive of high sperm DNA fragmentation, with negative, positive, and overall predictive values of 72.4%, 76.9%, and 74.5%, respectively. A receiver operating characteristic (ROC) curve using the logistic model presented an area under the curve (AUC) of 79.7% (p = 0.00002) (Fig. 2A). The highest sensitivity/specificity achieved was of 73.5% sensitivity and 77.8% specificity. ROC curves were also generated for raw values of each of the three significant proteins (MMP-2, MMP-7, and TIMP-4), with AUC values of 62.5% (non-significant – p = 0.073), 71.1% (p = 0.002), and 73.4% (p = 0.001), respectively (Fig. 2B). The highest sensitivity/specificity achieved was of 80.6% sensitivity and 44.1% specificity for MMP-2, 72.2% sensitivity and 67.6% specificity for MMP-7, and 86.1% sensitivity and 58.8% specificity for TIMP-4.

## Discussion

Seminal plasma is a complex secretion that contains many proteases originating either from the testes or from accessory sex glands^27,28^. Specifically, matrix metalloproteinases (MMPs) and tissue inhibitors of metalloproteinases (TIMPs) have been demonstrated to participate in mechanisms of human seminal plasma^2–4,29^. Previous studies have observed MMP-2, MMP-9^1,4,29^, TIMP-1 and TIMP-2 in human seminal plasma^10,12^. However, participation of other MMPs and TIMPs in human seminal plasma, and their relationship with sperm quality, remain to be elucidated.

This study set out to verify seminal levels of MMP-1, 2, 7, 9 and 10, and TIMP-1, 2, 3 and 4 in men with high versus low sperm DNA fragmentation, under the hypothesis that seminal MMPs and TIMPs associate to sperm DNA fragmentation, because matrix remodeling proteins have been associated with semen quality^30,31^. It was also hypothesized that differentially expressed proteins would predict DNA fragmentation status. Towards that end, a multiplex MAGPIX analysis was employed, using the Luminex xMAP technology. This technology has been demonstrated to present lower inter-assay variability than a traditional ELISA immunoassay, and results obtained by this assay are comparable to those obtained by a ELISA^32–34^.

MMPs are members of the metzincins family – a family of zinc-dependent proteases that digest extracellular matrix (ECM) components^35^. However, while this is the main reported function for MMPs, these proteins have been shown to participate in release and activation of growth factors and cytokines^36^ and to control apoptosis in the human reproductive tract^37,38^. MMPs are classified as (i) gelatinases (mainly target type IV collagen fibers), (ii) stromelysins (target noncollagen molecules), (iii) collagenases (target fibrillar collagen), and (iv) membrane-type (MT) MMPs (transmembrane enzymes that cleave ECM components and activate other MMPs)^35^.

MMPs were previously detected in the rat Sertoli cell^39^ and accessory sex glands^40^. The mechanism to control MMPs activation is complex and includes regulation of gene expression, cleavage of latent forms, and inhibition of active MMPs by their endogenous inhibitors: TIMPs^35^. In this study we did not perform relationship tests to compare levels of each studied MMP and their specific TIMPs because while TIMPs induce loss of proteolytic activity in their respective MMPS^1–3^, MMPs levels are not altered by TIMPs, only their function. MMPs may participate in seminal liquefaction after ejaculation with other proteinases, such as PSA^4^, although this may be only a part of their role in semen.

In this study, matrix metalloproteinase-2 (MMP-2) levels were decreased in seminal plasma of men with high sperm DNA fragmentation, with a low to medium effect size. This protein has been previously described in human seminal plasma^1,3,29,30^. MMP-2 activity was found in prostatic secretions of benign hyperplasic tissue^28,40^ and in sperm lysates^1^. MMP-2 was also observed in diverse cancers, such as breast, brain, ovarian, pancreas, colorectal, bladder, prostate and lung cancer^41–46^. MMPs are necessary for clearance of inflammatory cells in tissues^17^. As our hypothesis is that there is an increased inflammation in varicocele, and other cases of male infertility^23,26,47–54^, decreased levels of this protein could be related to increased levels of inflammation, due to the reduction in the clearance of inflammatory cells in the male reproductive system.

MMP-7 is a member of the stromelysin subfamily, with functions of degrading fibronectin, laminin, elastin, collagen, and proteoglycans^38^. MMP-7 was decreased in samples of men with high sperm DNA fragmentation. This protein cleaves Fas ligand (mFasL) to a functional soluble form (sFasL)^37^ and might have a function of regulating the Fas system in the reproductive tracts^38^. Moreover, there is evidence that deregulation of sFasL/MMP-7 production is related to decreased fertility^38^. The association of MMP-7 with control of apoptosis may explain the lower levels of this protein in the seminal plasma of the high sperm DNA fragmentation group, indicating that this group may present failure in apoptosis mechanism within the testes. With a medium to high effect size, this protein is likely involved in mechanisms of sperm DNA fragmentation.

TIMPs – endogenous inhibitors of MMPs^55–57^ – are described as possessing 4 family homologous members (TIMP-1, 2, 3 and 4)^55–57^. As a general rule, all TIMPs are capable of inhibiting all MMPs, but the efficacy of MMP’s inhibition varies for each TIMP^56,57^. The fact that TIMP-1 and 2 levels were decreased in semen of patients with high sperm DNA fragmentation strengthens results that point towards increased inflammatory status in the reproductive system/semen of men with causes for male infertility. A previous study using a mouse knock-out of TIMP-1 showed it controls ECM proteolysis^58^. In that study, the authors verified that this protein is related to preservation of a normal myocardial structure and function, and that it attenuates degradation of the extracellular matrix. Still, in pulmonary fibrosis there is an increased expression of TIMP-1, suggesting an important regulatory role for this protein in inflammatory and fibrotic responses^16^. Because inflammation was also increased in knockout mice, TIMP-1 is suggested as a restrainer of inflammation^19^. Its decreased levels may play a role in the molecular alterations that determine an inflammatory semen in male infertility.

TIMP-2 inhibits metalloproteinases, including MMP-2; however, this protein is also required for MMP-2 activation^59^. In this study, the high sperm DNA fragmentation group demonstrated lower levels of MMP-2 and TIMP-2, when compared with low sperm DNA fragmentation group. It may be that TIMP-2 in decreased levels compels MMP-2 towards decreased activity – which in turn leads to seminal inflammation and alteration in the clearance of inflammatory cells^15^. Finally, TIMP-4 was also decreased in seminal plasma of men with high sperm DNA fragmentation, with a medium to high effect size. This protein restricts ECM proteolysis in a number of tissues, including the female reproductive tract^60–63^. Knockout mice for TIMP-4 present increased interstitial fibrosis following injury^20^, which was associated with higher MMP-14 activity and increased inflammation, suggesting that TIMP-4 also regulates ECM deposition through inhibition of MMP-14 and restriction of inflammation^20^.

These results are further supported by the logistic regression analysis - a stepwise model was constructed by adding proteins with the highest likelihood ratio to improve the model, until no more significant proteins could be added. The final model included proteins MMP-2, MMP-7, and TIMP-4, and achieved an overall predictive value of 74.5%. The fact that individual values of MMP-7 and TIMP-4 were also able to separate low and high sperm DNA samples, as seen in Fig. 2B, further supports the potential for a non-invasive analysis of sperm DNA fragmentation in a sample – which could in the future lead to improved in-lab testing for sperm functional quality.

The results presented in this article are especially important in light of the fact that current diagnostic techniques for DNA fragmentation are conducted on sperm, and lead to their irreversible damage^64–66^. A quick test on surrounding seminal plasma would be non-invasive (in terms of sperm viability) and allow for rapid determination of DNA quality. Nevertheless, to use this protein as biomarker, more studies need to be performed in a larger cohort and using data of different DNA fragmentation tests, but this study clearly shows an advance in the comprehension of molecular mechanisms related to inflammation and sperm DNA fragmentation in seminal plasma samples.

Our results support the hypothesis of infertility as an inflammatory seminal event. Previous studies from our group have demonstrated that inflammation is present in different causes of male infertility, such as in the adolescent varicocele (in the presence of decreased semen quality)^54^, smoking^50,52^, semen lipid peroxidation^51^, and in patients who have sustained spinal cord injury (SCI)^53^. Furthermore, male infertility has been related to neutrophil and macrophage infiltration in semen^67^. These cells lead to sperm DNA fragmentation by generation of oxidative stress, release of hydrolytic enzymes, and cytokine-induced apoptosis^68,69^. With all this in mind, it seems that alterations in MMPs and TIMPs levels in seminal plasma of men with high sperm DNA fragmentation are related to the increase in the inflammatory processes of semen.

One of the limitations of this study is that sperm DNA fragmentation was diagnosed in a general population seeking conjugal infertility treatment, so that it is not possible to know if sperm DNA damage was generated during spermatogenesis, epididymal maturation, or post-ejaculation. Our results, thus, apply to a general human population, but stratification of these results according to origin of DNA damage should be verified in experimental models. Moreover, while multiplex protein expression analysis has been performed in seminal plasma^70^, a specific multiplex analysis of MMPs and TIMPs has not yet been published.

**Figure 3:**
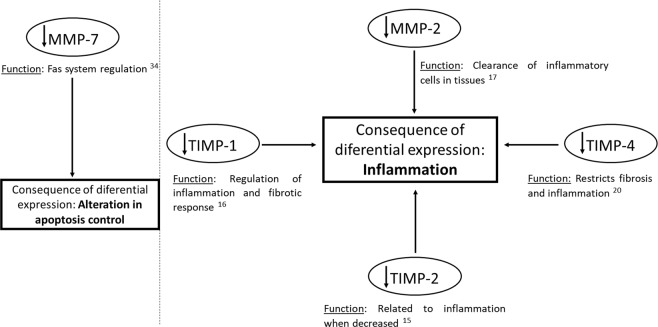
Probable MMP- and TIMP-associated mechanisms involved in sperm DNA fragmentation and inflammation.

To our knowledge, this is the first article to present multiplex data on extracellular matrix proteins in seminal plasma of adults. This has allowed us to propose the mechanism presented in Fig. 3: decreased levels of MMP-7 leading to apoptosis deregulation, MMP-2, leading to alteration in the clearance of cells related to inflammation in the male reproductive system, TIMP-1, TIMP-2, and TIMP-4, all of which increase inflammation. In conclusion, we have here demonstrated that MMP-2, MMP-7, TIMP-1, TIMP-2, and TIMP-4 levels are lower in semen of men with high sperm DNA fragmentation. We suggest this leads to a seminal inflammatory profile. We also suggest that monitoring MMP-2, MMP-7 and TIMP-4 may be a non-invasive method for determining the inflammation status of the semen, which can lead to altered fertility.

## Material and Methods

### Study design

A prospective study was carried out, including men recruited from the Andrology laboratory of the Human Reproduction Section (UNIFESP – Universidade Federal de Sao Paulo; Brazil) during investigation for conjugal infertility and volunteers who wished to participate in our study. Institutional Review Board approval was obtained from the Sao Paulo Federal University (UNIFESP; Brazil) Research Ethics Committee (CAAE: 54541516.2.0000.5505), and all included subjects provided their informed, written consent. All experiments were therefore performed in accordance with Brazilian federal guidelines and regulations for research projects involving human subjects. Inclusion criteria were men aged 20 to 50 years old and normal semen analysis according to the World Health Organization Guidelines^22^. Exclusion criteria were: fever in the 90-day period prior to semen analysis, presence of systemic diseases (such as cancer and endocrinopathies and their treatments), endocrine disorders, obesity, smoking, congenital malformation of the genitalia, genetic syndromes, prior history of inguinoscrotal surgery, orchitis or epididymitis, testicular torsion and testicular dystopia^71^.

Initially, 156 adults were recruited for semen and sperm functional analysis, as described below. Patients were then ranked by sperm DNA fragmentation, and the top and low 25^th^ percentiles were used to form the high and low (control) DNA fragmentation groups, respectively. The final number of samples used for evaluation of TIMPs and MMPs levels was 78 (n = 40 in low sperm DNA fragmentation group and n = 38 in high sperm DNA fragmentation group).

Semen samples were collected at the Andrology Laboratory of the São Paulo Federal University (Brazil), by masturbation after 2 to 5 days of ejaculatory abstinence^22^. After semen liquefaction, an aliquot was used for semen analysis, performed according to the WHO Guidelines^22^. Another aliquot was used for sperm DNA fragmentation, mitochondrial activity, and acrosome integrity analyses, and the remaining volume was centrifuged at 800 × G for 30 minutes to separate the supernatant seminal plasma, which was frozen without cryoprotectants and kept at −20 °C until MMPs and TIMPs levels analyses.

### Sperm DNA fragmentation analysis

Sperm nuclear DNA fragmentation was evaluated by a modified alkaline single-cell gel electrophoresis, or Comet assay as previously reported^49,70,71^. Slides were stained with SYBR Green (SYBR Green II RNA gel stain), diluted 1:10,000 (vol/vol) in TBE (0.1 M Tris [GE Healthcare, Amersham Place, UK]; 0.083 M boric acid; 0.001 M Na2- ethylenediaminetetraacetic acid [Carlo Erba Reagents, Cornaredo, Italy]) for 40 minutes, and washed with TBE to remove background staining.

A total of 100 sperm were analyzed using an Olympus BX-51 epifluorescence microscope, under 400× magnification, and Komet 6.0.1 software (Andor Technology) was used to assess sperm DNA fragmentation variables. The median of the Comet Distributed Moment variable, calculated by the software for each cell, was used as a marker of DNA fragmentation^72^. The Comet Distributed Moment is calculated according to comet length and fluorescence intensity and does not differentiate comet head from tail^72^.

### Sperm mitochondrial activity

Sperm mitochondrial activity was evaluated by midpiece sperm staining with 3,3′-diaminobenzidine (DAB)^23,50,52,73–80^, which is oxidized by the mitochondrial cytochrome C complex and accumulates in active mitochondria in the sperm midpiece^80^.

A total of 200 sperm were analyzed using an Olympus BX-51 phase contrast upright microscope (Olympus Corporation, Tokyo, Japan) under 1,000× magnification. Sperm cells were classified as: class I = 100% of the midpiece stained; class II = more than 50% of the midpiece stained; class III = less than 50% of the midpiece stained; and class IV = absence of staining in the midpiece^23,50,52,73,76^.

### Sperm acrosome integrity

Acrosome integrity was verified by staining the sperm with peanut agglutinin, a lectin that binds to the outer sperm acrosome membrane^23,52,81^. Briefly, two 15 μL smears were prepared on microscope slides and air dried. The slides were fixed in methanol (Merck Millipore, Massachusetts, USA) for 15 minutes and air dried again. Sperm were stained with 60 μg/mL fluorescein isothiocyanate (FITC)-conjugated peanut agglutinin (Sigma Aldrich, St. Louis, Missouri – EUA) in phosphate-buffered saline for 30 minutes in the dark and subsequently washed with Milli-Q water to remove background staining.

A total of 200 sperm were analyzed using an Olympus BX-51 epifluorescence microscope with appropriate filters (excitation of 494 nm wavelength, emission of 512 nm wavelength), under 1,000× magnification. Individual spermatozoa were classified according to acrosome integrity (intact, when fully stained, or damaged).

### Multiplex protein expression analysis

Multiplex protein expression analyses were carried out using a MAGPIX system (Merck Millipore, Billerica, USA). Initially, seminal plasma samples were thawed at room temperature and immediately centrifuged at 16,100 × G for 30 minutes at 4 °C in order to remove cellular debris. Supernatants were then collected. Total protein concentration of each seminal plasma sample was measured using a modified Lowry - Bicinchoninic Acid (BCA) assay, according to the manufacturer’s recommendation^82^. Samples were diluted (1:80) in milli-Q water and measured in triplicate and standard curve points (0, 200, 400, 600, 800, and 1,000 μg/mL of bovine serum albumin in water) were measured in duplicate in a 96-well plate. Absorbance was measured using a microplate reader at 540 nm. Samples with a coefficient of variation of over 5% were re-quantified, to ensure accurate quantification.

Two protein biomarker panels of a 96-well plate each were used: (1) Milliplex Human MMP panel consisting of MMP-1, MMP-2, MMP-7, MMP-9, and MMP-10 (Millipore kit no. HMMP2MAG-55K); and (2) Milliplex Human TIMP panel consisting of TIMP-1, TIMP-2, TIMP-3, and TIMP-4 (Millipore kit no. HTMP2MAG-54K). Protocols were followed according to the manufacturer’s instructions. All buffers used are proprietary components of these kits and the method followed the manufacturer’s recommendations. For each plate, we prepared a standard curve based on serial dilutions and each sample was previously diluted in assay buffer (to a final concentration of 3 μg/mL for MMPs and 12 μg/mL for TIMPs).

Following the addition of beads, the MMPs plate was incubated on a shaker at room temperature for 2 hours and the TIMPs plate was incubated overnight on a shaker at 4 °C. After this incubation, both plates were washed twice with wash buffer. Then the detection antibodies were added into all plates’ wells, and both plates were incubated on a shaker for 1 hour at room temperature. After this period, streptavidin-phycoerythrin were added into all plates’ wells and incubated with agitation on a shaker for 30 minutes at room temperature. The plates were then washed twice, and drive fluid (MAGPIX®) was added to all wells. The plates were kept in a shaker at room temperature for 5 minutes, to resuspend the beads, and were then read using MAGPIX® (Millipore) with xPONENT software.

### Statistical analysis

Statistical analyses were performed using SPSS 18.0 (PASW) for Windows. Semen and sperm functional analyses and multiplex protein expression data were tested for normality and homoscedasticity using a Kolmogorov-Smirnov test and a Levene test, respectively. One extreme value was removed from MMP-1 samples, and three from MMP-9. MMP-9 values were also log-transformed in order to achieve normality of distribution. Groups were compared using an unpaired Student’s T test. Effect size was assessed using Cohen’s coefficient, which normalizes differences between group means to their standard deviation^83^. As per Cohen’s interpretation, effect size was considered low when below 0.25, medium when d = 0.5, and high when d = 0.8^83^.

In order to assess predictive value of these proteins, a logistic regression model was constructed, using a forward stepwise likelihood method – independent variables (protein concentrations for each of the proteins) were added to the model based on the highest likelihood ratio for improving prediction of group (dependent variable – low or high sperm DNA fragmentation), until addition of another variable would not improve the model. Negative, positive, and overall predictive values for this logistic model were reported. A Receiver Operating Characteristics curve was constructed, both for the logistic model itself and for each of the untransformed values of proteins which were significant in the model. Results were considered significant when p < 0.05, and a maximum beta error was set at 20% (minimum power = 0.8).
