# Author Correction: Semen levels of matrix metalloproteinase (MMP) and tissue inhibitor of metalloproteinases (TIMP) protein families members in men with high and low sperm DNA fragmentation

**DOI:** 10.1038/s41598-019-45361-2

**Published:** 2019-07-10

**Authors:** Larissa Berloffa Belardin, Mariana Pereira Antoniassi, Mariana Camargo, Paula Intasqui, Renato Fraietta, Ricardo Pimenta Bertolla

**Affiliations:** 1 Department of Surgery, Division of Urology, Universidade Federal de São Paulo, São Paulo, Brazil; 2 Hospital São Paulo, São Paulo, Brazil

Correction to: *Scientific Reports* 10.1038/s41598-018-37122-4, published online 29 January 2019

The original version of this Article contained an error in the title of the paper, where the word “metalloproteinases” was incorrectly given as “metallorproteinases”. This has now been corrected in the PDF and HTML versions of the Article.

